# Classification of large circulating tumor cells isolated with ultra-high throughput microfluidic Vortex technology

**DOI:** 10.18632/oncotarget.7220

**Published:** 2016-02-06

**Authors:** James Che, Victor Yu, Manjima Dhar, Corinne Renier, Melissa Matsumoto, Kyra Heirich, Edward B. Garon, Jonathan Goldman, Jianyu Rao, George W. Sledge, Mark D. Pegram, Shruti Sheth, Stefanie S. Jeffrey, Rajan P. Kulkarni, Elodie Sollier, Dino Di Carlo

**Affiliations:** ^1^ Department of Bioengineering, University of California, Los Angeles, California, USA; ^2^ Vortex Biosciences, Menlo Park, California, USA; ^3^ Department of Surgery, Stanford University School of Medicine, Stanford, California, USA; ^4^ Department of Hematology & Oncology, UCLA Medical Center, Los Angeles, California, USA; ^5^ Department of Pathology & Laboratory Medicine, UCLA Medical Center, Los Angeles, California, USA; ^6^ Stanford Women's Cancer Center, Stanford, California, USA; ^7^ Division of Dermatology, UCLA Medical Center, Los Angeles, California, USA; ^8^ California NanoSystems Institue, Los Angeles, California, USA; ^9^ Jonsson Comprehensive Cancer Center, Los Angeles, California, USA

**Keywords:** circulating tumor cells, immunofluorescent staining, rare cell enrichment, size based cell isolation, Vortex

## Abstract

Circulating tumor cells (CTCs) are emerging as rare but clinically significant non-invasive cellular biomarkers for cancer patient prognosis, treatment selection, and treatment monitoring. Current CTC isolation approaches, such as immunoaffinity, filtration, or size-based techniques, are often limited by throughput, purity, large output volumes, or inability to obtain viable cells for downstream analysis. For all technologies, traditional immunofluorescent staining alone has been employed to distinguish and confirm the presence of isolated CTCs among contaminating blood cells, although cells isolated by size may express vastly different phenotypes. Consequently, CTC definitions have been non-trivial, researcher-dependent, and evolving. Here we describe a complete set of objective criteria, leveraging well-established cytomorphological features of malignancy, by which we identify large CTCs. We apply the criteria to CTCs enriched from stage IV lung and breast cancer patient blood samples using the High Throughput Vortex Chip (Vortex HT), an improved microfluidic technology for the label-free, size-based enrichment and concentration of rare cells. We achieve improved capture efficiency (up to 83%), high speed of processing (8 mL/min of 10x diluted blood, or 800 μL/min of whole blood), and high purity (avg. background of 28.8±23.6 white blood cells per mL of whole blood). We show markedly improved performance of CTC capture (84% positive test rate) in comparison to previous Vortex designs and the current FDA-approved gold standard CellSearch assay. The results demonstrate the ability to quickly collect viable and pure populations of abnormal large circulating cells unbiased by molecular characteristics, which helps uncover further heterogeneity in these cells.

## INTRODUCTION

Circulating tumor cells (CTCs) are cancer cells that have been shed from a tumor into the bloodstream and play a major role in metastasis. The relative number of CTCs is predictive of patient prognosis and treatment efficacy [1, 2]. Moreover, there is growing interest in using CTCs as non-invasive cellular markers of cancer genotypic and phenotypic changes for both clinical and research applications, and so it is important to be able to isolate and analyze CTCs from a vial of patient blood. Nevertheless, CTCs are extremely rare and are found at concentrations as low as 1-10 CTCs/mL of whole blood in a background of millions of white blood cells (WBCs) and billions of red blood cells (RBCs).

Many current technologies employ affinity-based capture methods, using antibodies or aptamers that bind to cell surface markers [3]. The CellSearch system (Janssen Diagnostics) is the current gold standard prognostic tool which makes use of a ferromagnetic immunoaffinity assay that targets CTCs using probes against epithelial cell adhesion marker (EpCAM) [4, 5]. Despite achieving high capture efficiencies using cultured cell lines, it remains challenging to capture heterogeneous patient sample CTCs, many of which are now known to undergo an epithelial-to-mesenchymal transition (EMT) which may involve downregulation of target epithelial cell surface markers [6, 7]. Alternative technologies are emerging which target cell size as an alternative biophysical marker for CTCs. For example, the ISET [8-10] and ScreenCell [11, 12] devices make use of porous filters to separate larger CTCs from the smaller RBCs and WBCs, and more recent microfluidic approaches further refine the interaction between cells and microfabricated structures [13, 14], including the use of microchannel constrictions [15], micropillar arrays [16], and other microfilter variations [17-19]. Nevertheless, direct filtration approaches are prone to clogging due to intrinsic interactions between sticky cancer and blood cells and filter surfaces, and cells may be difficult to release for further downstream analysis.

Continuous flow microfluidics has emerged as a promising technology for the reduced-contact isolation and extraction of viable CTCs by size, such as hydrodynamic filtration [20, 21] or deterministic lateral displacement [22, 23]. Recent advances in inertial microfluidics have offered a more rapid platform through which cells may be sorted by size, as fluidic forces generated from high flow rates scale strongly with cell size [24, 25]. Notable technologies with high capture efficiencies include the use of shear gradient lift forces in expansion-contraction microchannels [26-28], Dean flow fractionation (DFF) in spiral microfluidic chips (Clearbridge Biomedics) [29, 30], and inertial focusing prior to WBC negative depletion using immunomagnetic beads (CTC-iChip) [31]. Still, current techniques have been hindered by scalability, low sample purity, and dilute output sample volumes which require additional cell concentration steps.

Although size-based isolation and negative depletion approaches may acquire subpopulations of cells that have undergone EMT or other trans-differentiation processes, collected cells can be difficult to identify with commonly used stains (e.g. cytokeratins, CK) optimized for cells of epithelial origin. Studies have found irregular CTC expression profiles in which epithelial (CK, EpCAM), mesenchymal (vimentin, N-cadherin), or potentially either both or neither markers are expressed [32-34]. Additionally, non-specific binding of probes may result in cross-reactivity and cause difficulties in proper cell identification. Although the CellSearch CellTracks Analyzer II semi-automated system aids in CTC identification, identification is dependent solely on CK expression, and there remains variability of cell classification between trained operators [4, 5]. Finally, variability of staining protocols, antibody clones between vendors, and imaging setups causes conflicting definitions of CTCs. A more general, standardized staining and classification approach is required, which takes into account cells that are negatively- or doubly-stained for standard markers. Cytomorphological characteristics, such as abnormal cell size and large nuclear-to-cytoplasmic (N:C) ratios, are also indicators of malignancy or hematopoietic origins [35] that may be factored in with high quality imaging for cell identification. Because of differences in implementing immunofluorescence staining and counting protocols between labs, an ideal classification approach should be rigorously described and demonstrated with galleries of images and detailed training documents.

We have previously described the Vortex Chip [36], a simple microfluidic device with rectangular reservoirs which generate stable laminar fluid microvortices at high flow rates to passively trap, purify, and concentrate large CTCs from blood or other body fluids [37]. Despite achieving high purity, the previous device was limited by low capture efficiency (∼20%) and a high but un-optimized throughput for processing 10x diluted blood (4 mL/min). Here, we introduce the High Throughput Vortex Chip (Vortex HT), which demonstrates markedly increased processing speed and tunable capture efficiency achieved by reflowing of the sample waste. We also implement objective, standardized classification criteria which are explained and demonstrated with detailed protocols and image galleries from new clinical studies, and use these criteria to compare device performance directly with previous designs and the gold standard for the same cancer patients. Vortex HT outperforms previous Vortex designs and the gold standard CellSearch system over a range of criteria, yielding a flexible and high-performance technology for obtaining CTCs for clinical and research purposes.

## RESULTS

### Design iterations toward Vortex HT

**Figure 1:**
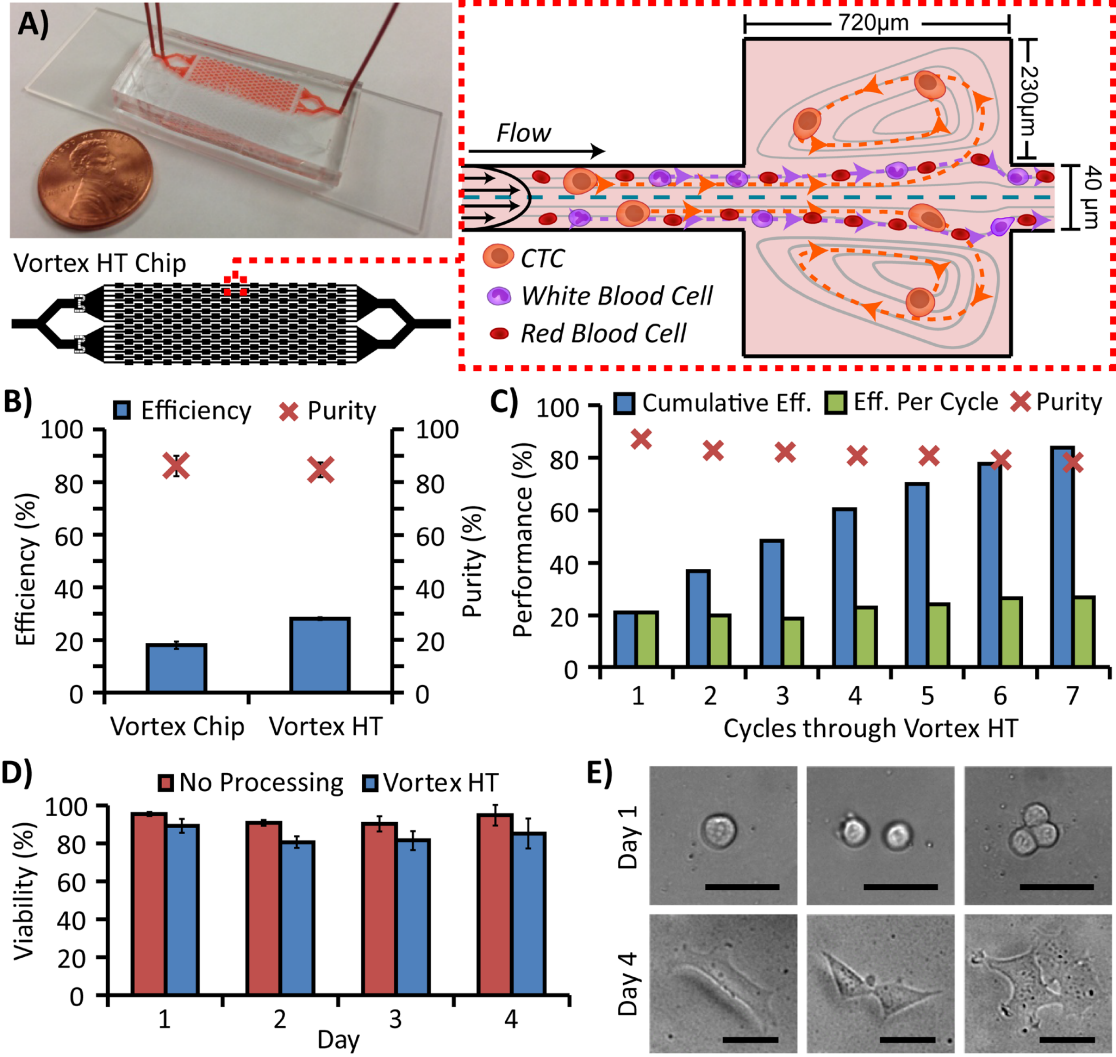
Microfluidic device design and performance **A.** The High Throughput Vortex Chip (Vortex HT) is parallelized with 2x more parallel channels and 1.5x more serial reservoirs in each channel than the previous Vortex Chip design. At high flow rates, microvortices develop in each reservoir and trap larger cancer cells while allowing smaller RBCs and WBCs to either pass through or transiently enter vortices. **B.** With the same processing time, Vortex HT yields ∼1.6x higher capture efficiency of MCF7 breast cancer cells while maintaining comparable purity (*n* = 3 trials) relative to the Vortex Chip. **C.** Sample flow-through may be collected and repeatedly processed through multiple cycles to increase cell capture with a tradeoff of slightly diminished sample purity. **D.** MCF7 cells processed through Vortex HT maintained high relative viability compared with cells not processed through the device. **E.** MCF7 cells released into a well-plate are able to grow and proliferate over the course of 4 days. Scale bar represents 40 μm.

While previous work has assessed the effect of sample concentration, biophysics, and other extrinsic factors on the efficiency of Vortex trapping [36], the Vortex HT design—used throughout the study (Figure 1A)—was established after several design iterations (Supplementary Figure 1) which explored the intrinsic device layout and maximized the capture potential and throughput of Vortex trapping. First, the long straight upstream channel of the original Vortex Chip was found to be unnecessary for trapping. While a 10,000 μm upstream length was initially thought to provide inertial cell focusing toward the two lateral side walls and in closer proximity to the vortex region to improve capture, COMSOL software simulations demonstrated that the fluid flow profile fully develops within a relatively shorter 500 μm minimum distance (Supplementary Figure 2), suggesting that sufficient shear gradient lift forces are achievable over shorter distances for cells already present near walls. Tests revealed a peak efficiency with a 500 μm upstream distance before reservoirs, below which efficiency decreased. A 1000 μm straight channel distance between reservoirs was chosen to achieve a balance between high efficiency and purity (Supplementary Figure 2D). Accordingly, replacement of the long straight upstream focusing channel in the Vortex Chip with serial 1000 μm spaced reservoirs was found to improve cell capture. High fluidic resistance and risk of device failure and delamination limited further serialization of reservoirs, restricting the design to 12 reservoirs per channel. Next, parallelization from 8 to 16 channels enabled a 2X faster flow rate while maintaining the same Reynolds number, the ratio of inertial to viscous forces in the flow, necessary for capture. When characterizing trapping from a polydisperse solution of deformable PDMS beads, Vortex HT demonstrates selective enrichment for particles greater than ∼13 μm (Supplementary Figure 3).

### Device performance with cell lines

With the reduced time to process a sample using Vortex HT, saved time may be used to reprocess the fluid waste from the first trapping cycle to achieve higher capture efficiency for cancer cells. In the same processing time as the Vortex Chip, Vortex HT recovers cells at 1.6x higher efficiency using 2 cycles of processing (Figure 1B) while maintaining high sample purity (>80%). Recovery is further enhanced by multiple rounds of reprocessing (with a trade-off of increased run time), resulting in up to 84% cumulative efficiency after 7 processing cycles of 4 mL of 10x diluted blood spiked with 300 MCF7 cells (Figure 1C). Interestingly, taking into account the remaining non-captured cells that are infused through the device, the adjusted efficiency per cycle was comparable for all cycles (avg. 22.8% ± 3.2%), suggesting that the entry and maintenance of cells in vortex traps is a probabilistic random process which may follow Poisson statistics. Consequently, sample reprocessing is reproducible between cycles and devices (Supplementary Figure 4). The captured cells remain viable and may be collected directly off-chip—in a concentrated ∼150 μL volume per cycle—and cultured for over 4 days (Figure 1D-1E). Viability from Vortex HT remained even throughout the study (avg. 83.9% ± 4.0%, with an avg. 8.8% ± 1.8% lower viability from the control) and slightly increased by day 4, which may be due to the increase in number of proliferated cells.

### CTC classification criteria

**Figure 2:**
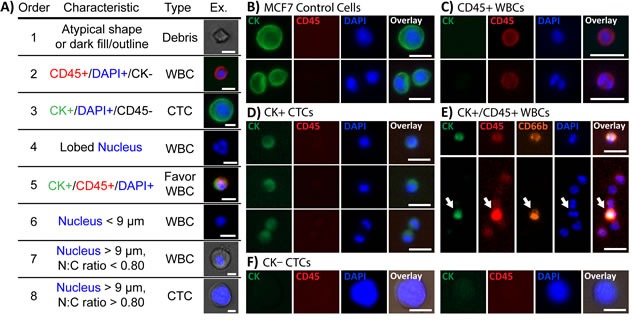
Immunofluorescent staining characteristics for identifying CTCs **A.** Collected cells were classified according to immunostains against CK (green) and CD45 (red), and DNA stained with DAPI (blue). In general, CTCs were defined as either CK+/CD45−/DAPI+ or only DAPI+ with a large nucleus (>9 μm) and N:C ratio (>0.8). Each cell was compared with the table's criteria in the order listed until the characteristics matched. Scale bars represent 10 μm. **B.** MCF7 cells, used as a staining control, stained strongly for CK and negative for CD45. **C.** Most WBCs stained for CD45-TRITC but negative for CK. Monocytes and lymphocytes were consistently stained strongly whereas granulocytes exhibited weaker CD45 signals. **D.** CTCs typically stained weakly or negatively for CK. **E.** Several cells stained double-positive for both CD45 and CK. Additional staining of the collected cells with CD66b-AF647 confirmed the cells as activated granulocytes. **F.** Large CK−/CD45- cells with high N:C ratios were present in collected samples and defined as CTCs by our criteria. [All scale bars in (B-F)]represent 20 μm.

Having cells freely released into solution and collected on optimized optical substrates without interfering bound beads allows for high quality fluorescence and brightfield imaging that reveals morphologies unique to CTCs. Based on standard CK and CD45 immunostaining and morphological features that are diagnostic in cytopathology, a set of criteria was developed to classify cells (Figure 2A), based on both existing methods [35, 38, 39] and observations of cell populations captured from healthy donor samples using Vortex HT. Classifications were comprised of 3 categories: debris, WBCs, or CTCs. In general, debris was characterized by irregular, jagged shapes or dark outlines under bright-field microscopy. Aside from the clear distinctions of CTCs as CK+/CD45−/DAPI+ (Figure 2B, 2D) and WBCs as CK−/CD45+/DAPI+ (Figure 2C), incidences arise in which cells may be doubly-stained (CK+/CD45+/DAPI+) or only DAPI+. Staining with CD66b confirmed that doubly-stained cells corresponded to activated granulocytes (Figure 2E), and were thus classified as WBCs. For instances in which cells stained only DAPI+, WBCs were distinguished by lobular or segmented granulocytic nuclei, small nuclei (<9 μm), and/or small N:C ratios, defined in this study as the imaged area of the nucleus to the area of the surrounding cytoplasm. CTCs were primarily characterized morphologically by a large nucleus (> 9 μm) and large N:C ratio (Figure 2F). Detailed explanations of cell classifications with accompanying image galleries and training worksheets are included as Supplementary Material.

### Device performance with clinical samples

**Figure 3:**
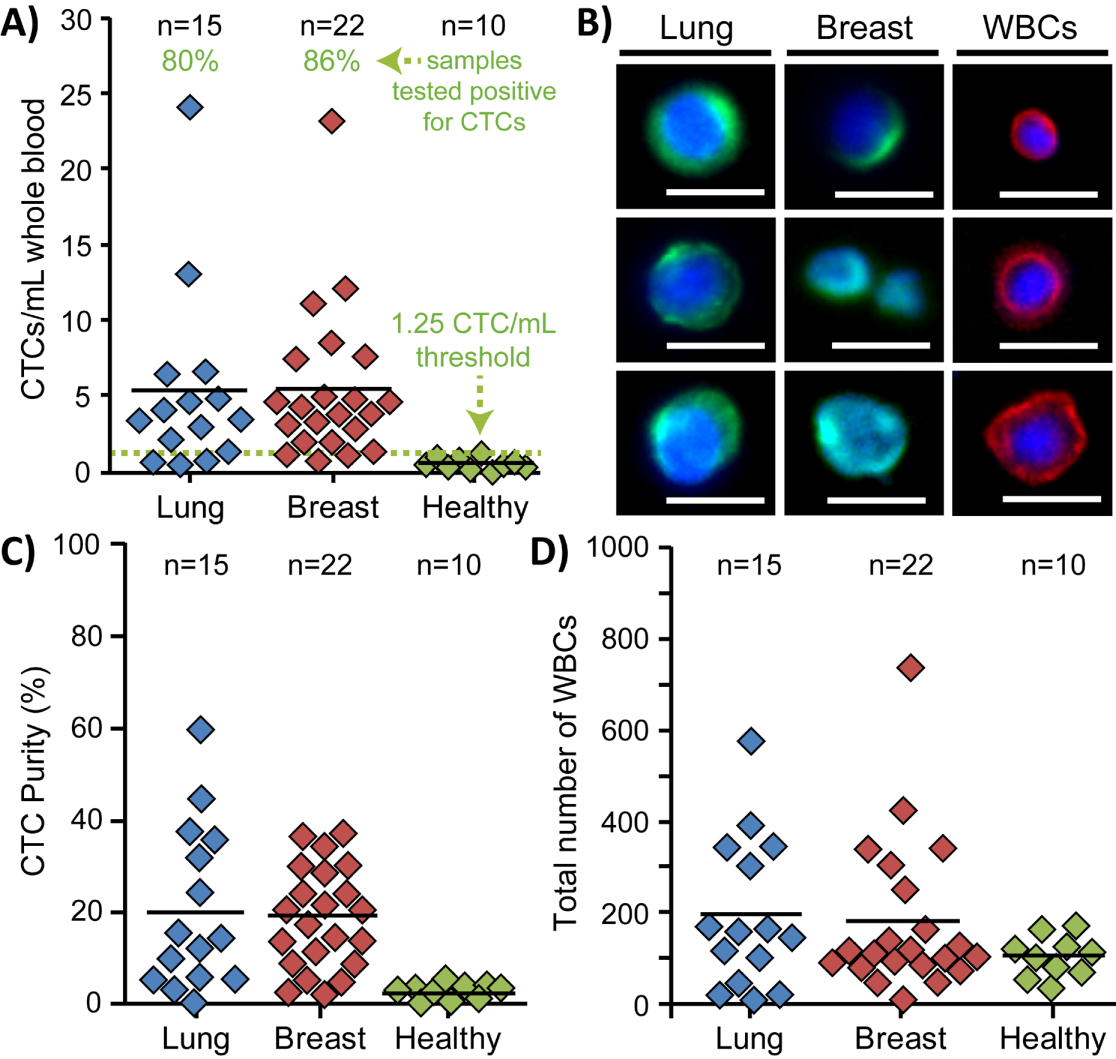
Enumeration of patient CTCs **A.** More CTCs/mL of whole blood were found in stage IV lung (*n* = 15) and breast (*n* = 22) cancer blood samples than in age-matched healthy samples (*n* = 10). Based on the maximum count for all healthy samples, a minimum threshold of 1.25 CTCs/mL of whole blood (dotted green line) was set to define samples as CTC-positive. Using such a threshold, approximately 80% and 86% of lung and breast cancer samples, respectively, were found positive for CTCs. **B.** Representative immunofluorescence images of CTCs and WBCs collected from Vortex HT. Scale bars represent 20 μm. **C.** The purity of collected CTCs varied between samples and averaged ∼20%. Healthy samples exhibited very low purity due to the few collected cells which were classified as CTCs. **D.** The absolute number of collected WBCs was relatively low as well and was present in all processed samples.

A total of 22 breast, 15 lung, and 10 age-matched healthy blood samples were used in the study (Supplementary Figure 5, Supplementary Table 1). The median age was 66 years (range 37-91), and the majority of cancer patients (36/37) were undergoing treatment at the time of draw. Using the classification criteria, more CTCs were found in lung (mean: 5.3 CTCs/mL, range: 0.5-24.2 CTCs/mL) and breast (mean: 5.4 CTCs/mL, range: 0.75-23.25 CTCs/mL) cancer samples than in healthy controls (mean: 0.56 CTCs/mL, range: 0-1.25 CTCs/mL) (Figure 3A). A low number of cells were characterized as CTCs in healthy samples, with a maximum count of ∼1.25 CTCs/mL, which is lower than that found from previous techniques [36] and suggests a higher achieved specificity using a combination of staining and morphological criteria. Using this baseline value as a threshold, approximately 80% and 86% of lung and breast cancer samples, respectively, were found to be positive for CTCs. Additionally, although lower than tests with cell lines, the purity of cancer blood samples remained quite high (avg. 19.8% ± 13.9%, Figure 3C), and up to an order of magnitude higher than competing technologies such as the CTC-iChip [40]. Moreover, the low absolute number of captured WBCs in cancer samples (avg. 187 ± 164, Figure 3D) represents an approximately 10^4^-10^5^ fold depletion, which enables greater signal-to-noise results in downstream analyses that are limited by the presence of interfering wild-type cells. Interestingly, the absolute number of WBCs appears to reveal a baseline value below 200 cells in all samples, with few outliers (4 lung and 6 breast samples), although it is unclear if the outliers are due to unique patient states which may magnify the quantity of WBCs captured in Vortex HT.

**Figure 4:**
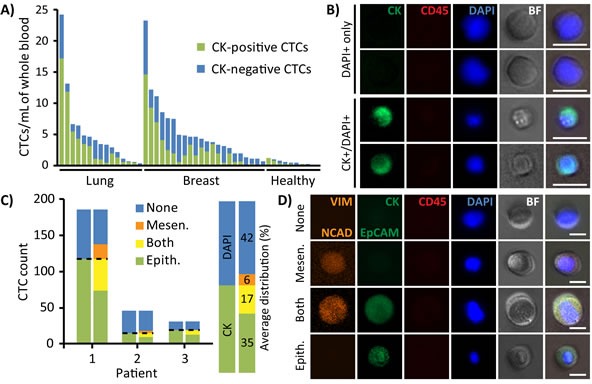
Immunofluorescent profiles of patient CTCs **A.** CTCs collected from each patient sample were composed of both CK-positive (green) and CK-negative (DAPI+ only, blue) subpopulations. **B.** Representative images of CK−/DAPI+ and CK+/DAPI+ stained CTCs. Scale bar represents 20 μm. **C.** After traditional CK staining (left bar), cells immunostained for epithelial (EpCAM and CK, green) and mesenchymal (VIM, orange) markers (right bar) exhibited diverse variations of all combinations, including expression of both EMT markers or neither. The majority of cells were VIM−/CK+/EpCAM+/DAPI+ or VIM−/CK−/EpCAM−/DAPI+. **D.** Representative images of cells which express mesenchymal markers, epithelial markers, neither, or both. Scale bar represents 20 μm.

Captured CTCs displayed varying levels of CK expression, with 40.8% of total CTCs not expressing CK at all (Figure 4A, 4B). Interestingly, this percentage was not reflected at the individual patient level, which exhibited a wide range of CK−/DAPI+ CTCs with percentages per patient ranging between 8.7-100% (mean 49.0 ± 24.2%). To explore the nature of CK- CTCs, some samples were additionally stained for EMT markers using antibodies against Vimentin (VIM), N-Cadherin (NCAD), and EpCAM (Figure 4C). A small fraction of CK−/DAPI+ cells (12.5%) stained positive for VIM/NCAD, representing an average 6% of all CTCs collected. Additionally, an average of 32.7% of CK+/DAPI+ cells stained positive for both epithelial and mesenchymal markers, representing an average 17% of all CTCs. Nevertheless, a large 42% of CTCs were still negative for all EMT markers and only stained positive for DAPI, suggesting that additional staining may not significantly supplement standard CK stains and cytomorphological characterization as outlined in Figure 2.

### Comparison of Vortex HT with other technologies

**Figure 5:**
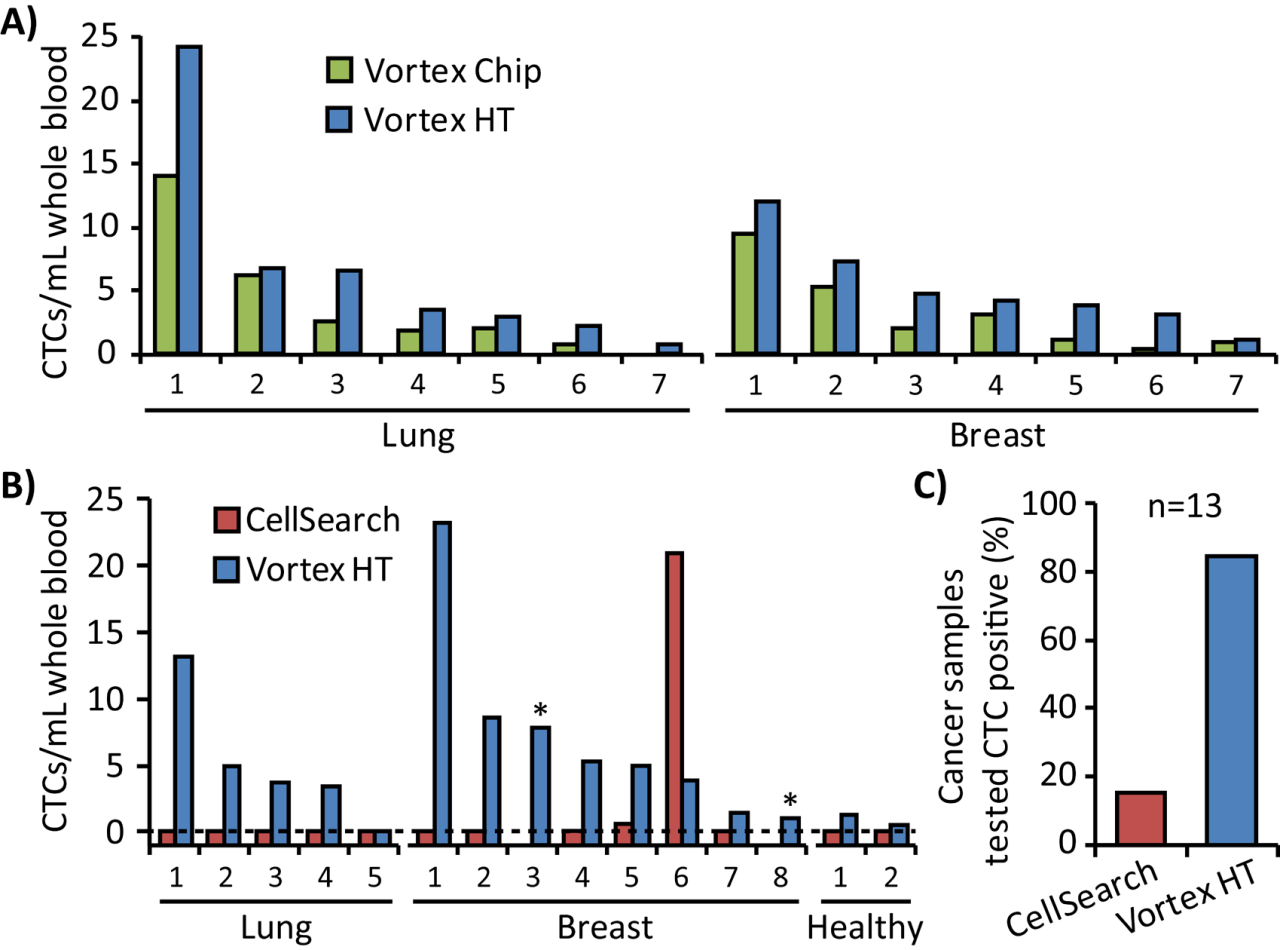
Comparison of Vortex HT with other technologies **A.** Vortex HT captures more CTCs than the Vortex Chip in all cases (7 lung and 7 breast). **B.** Blood tubes from the same patient were split for tests between Vortex HT and CellSearch for 5 lung, 8 breast, and 2 healthy samples. In two breast patients (no. 3 and 8, starred), the test was aborted by the CellSearch machine. **C.** In 13 of the metastatic cancer patient samples tested, the CellSearch test identified CTCs in 15% of samples, whereas Vortex HT found 85% of samples as positive for CTCs above levels for age-matched healthy controls.

Vortex HT enriched for a larger number of CTCs than the Vortex Chip in all 7 lung and 7 breast cancer patient samples tested, using the same volume of sample and same processing time for each device (Figure 5A). Notably, the numbers of CTCs captured with Vortex HT correlated with the number of CTCs isolated with the Vortex Chip (R^2^ =0.92, slope=1.44). That is, samples with larger CTC numbers captured by Vortex Chip had concomitantly larger capture numbers by Vortex HT. These results also demonstrated the chip-to-chip concordance of this processing approach, which suggests minimal variation induced by the capture technology itself. For 13 cancer patient samples tested with CellSearch, Vortex HT found 85% positive for CTCs above a healthy patient cut-off whereas CellSearch found only two samples (15%) positive above the healthy patient cut-off value for that system (Figure 5B-5C). Moreover, the number of CTCs captured in these two samples (breast sample no. 5 and 6, Figure 5B) were markedly different between CellSearch and Vortex HT, which is likely due to the differing selection parameters of EpCAM expression with CK positivity versus cell size and a combination of immunofluorescence and morphological features. No CTCs were detected from lung samples by CellSearch, which may be due their reduced expression of EpCAM, and supports the fact that CellSearch is only FDA approved for breast, prostate, and colon cancer blood samples. Two other samples (starred, Figure 5B) exhibited issues with the CellSearch instrument run by Quest Diagnostics, which displayed the error “Machine aborted sample during run”, and were deemed as uninterpretable for CTCs by the test system.

### Leukemia case

One healthy donor self-reported diagnosis with chronic myelogenous leukemia (CML) 20 days after having blood drawn for the study, and this patient was removed from the analysis of healthy samples. Interestingly, very large cytomorphologically atypical WBCs were found after processing with Vortex HT, before the patient was treated (Supplementary Figure 6). The cells were characterized by a range of N:C ratios, but all were over 20 μm in diameter and CD45+/DAPI+/CK−/CD66b−. An additional sample of blood was later acquired from the same patient during treatment. Complete blood counts (CBCs) of the patient showed a high concentration of WBCs (40.9 K/μL), far above the normal range expected in a healthy patient (4-11 K/μL), and later decreased to 3.1 K/μL while under treatment (Supplementary Figure 6C). In a similar trend, fewer atypical WBCs were captured from Vortex HT in the second draw. No such atypical white blood cells were seen in healthy donor samples, nor in lung or breast cancer patient samples, which may suggest the utility for Vortex HT as a general approach to enrich for other large circulating cells useful for the detection and analysis of other diseases.

## DISCUSSION

The simple geometry of Vortex HT, consisting only of straight microchannels and rectangular trapping regions, enables straightforward device fabrication and sample processing procedures. With minimal pretreatment steps that may damage cells, a vial of blood (∼8 mL) may be processed in 2 cycles within a short ∼20 min period, yielding demonstrably higher CTC counts in cancer patient specimens over healthy blood and a higher positive success rate compared with CellSearch. CellSearch found just one breast cancer sample above its defined 5 CTCs per 7.5 mL threshold that defines patients with poor prognosis and shorter survival time, which may suggest that the other samples (from patients undergoing treatment) were exhibiting low progression. While it is unclear if the baseline presence of cells classified as CTCs in healthy samples from Vortex HT arise from epithelial cell shedding from the blood draw puncture, nonspecific staining, or a baseline level normally found in the blood stream, the relatively low 1.25 CTCs/mL threshold still distinguished many patients as positive for CTCs. The consistent counts above the threshold from cancer samples may implicate Vortex HT as a more sensitive tool for detecting a diverse set of CTCs, which may yield broader distinctions in patient prognoses than CellSearch, but will first require longer-term studies with regular patient follow-up.

In relation to other competitive inertial microfluidic technologies, Vortex HT demonstrated slightly lower capture efficiencies than DFF (∼85%) and the CTC-iChip (∼95% with negative depletion), but it adds several advantages. First, the device hastens downstream analyses with its ability to concentrate cells from any volume to ∼200 μL, suitable for immunostaining and quick imaging in a single well of a 96-well plate, whereas competing technologies must either scan across a large area of a glass slide [40] or perform a centrifugation and resuspension in a smaller volume [29], which may lose rare CTCs in the process. Next, Vortex HT maximizes throughput, operating at the highest known reported flow rate (8 mL/min for ∼80 mL of 10x diluted blood), compared with ∼100 μL/min for 10 mL of whole blood using the CTC-iChip [40] and comparable to 350 μL/min for 3.75 mL of RBC-lysed blood using the modified high-throughput DFF [41]. Finally, the presented technology remains a top candidate in removing background cells, yielding half the number of WBCs as DFF [29] and over an order of magnitude greater purity than the CTC-iChip [40].

The high purity achieved with Vortex HT may facilitate CTC genotyping (sequencing, cytogenetics, etc.) in a step toward new drug discovery, personalized medicine, and informed treatment decisions for patients. Since cells also remain viable, Vortex HT enables other downstream analyses of live CTCs, including single-cell RT-PCR, cell culture, pharmacological studies [42], and single-cell Western blotting [43]. Moreover, the device provides a convenient sample preparation step that may be streamlined with cytopathology or immunocytochemistry techniques, in which technicians are often burdened by low sample purity. In addition to lung and breast cancer CTCs, the size-based isolation platform may potentially be applied for a variety of other cancer types (prostate, colon, melanoma, bladder cancer, etc.), or even other cell types (tumor cells, stem cells, endothelial cells, etc.) within a variety of biofluids (blood, urine, pleural [37] and peritoneal fluid, etc.).

The presence of atypical WBCs from a CML patient sample suggests that Vortex HT may also isolate large leukemic blasts. As CML cells range in size, with ∼35% of cells in the range of 14-35 μm [44], Vortex HT may be effective in purifying rare subpopulations of large cells which may otherwise remain hidden from affinity-based capture approaches. Although it remains unclear if the isolated cells are malignant cell precursors, immature white blood cells, or apoptotic cells, the absence of such cells from lung, breast, and healthy donor samples suggest their unique role in CML. These preliminary findings suggest further work is warranted to evaluate Vortex HT as an enrichment tool for a more sensitive identification of patient state that may be important for minimal residual disease monitoring. While relatively little microfluidic work has focused on sample preparation for observing and diagnosing CML, current techniques of isolation remain time-consuming or not fully developed [45]; Vortex HT may offer a high throughput, label-free means for leukemia cell purification. More broadly in a screening role, Vortex trapping from blood that yields an atypical large cell count may provide an earlier indication of a brewing disease process for a range of disease states [46], suggesting additional diagnostics to define the source of the large circulating cells may be warranted for the patient.

The objective cell identification criteria presented here addresses common but widely unreported concerns surrounding immunostains. Since many cells may transition to a mesenchymal state [32], traditional epithelial cell staining techniques may overlook a significant number of candidate cells [47], resulting in underreported performances especially in size-based isolation platforms. While most devices are characterized using probes for CK, CD45, and DAPI, the introduced CTC identification criteria makes use of a sequential checklist that includes well-defined morphological criteria associated with malignancy—which take advantage of accumulated cytopathology knowledge [10, 38, 39, 48]—and may help minimize user-errors in manual enumeration. Morphological characterization may also help classify large cells that stain negative for common CTC markers, which may arise from size-based isolation methods, and cytometric analyses may sufficiently distinguish CTCs from other cell types present in blood, like monocytes, granulocytes, and cancer-associated non-CTCs such as disseminated tumor-activated macrophages [49]. We expect the described cell identification protocol will complement future device performance characterizations, clinical applications, and help standardize existing commercial prognostic and sample preparation tools as well as those in development. To help others who wish to adopt these tools, we provide a comprehensive guide and training worksheets (Supplementary Material) to more effectively convey our accumulated knowledge. As with most available techniques, the introduced enumeration protocol is not fully comprehensive and does not factor in the use of other marker types, including those that are cancer origin-specific (e.g., anti-HER2 staining for breast cancer samples, or anti-PSA for prostate cancer). We expect that the presented criteria will help foster future discussions regarding thorough validation of CTCs, and envision that the described criteria can serve as a starting point for further adaptations to the method as promising new markers or automated imaging software become available.

## MATERIALS AND METHODS

### Device operation and fabrication

Vortex technology is a 70 μm-depth polydimethylsiloxane (PDMS) device with an array of 40 μm width straight channels, each leading to a series of 500 μm x 720 μm rectangular trapping reservoirs, spaced 1000 μm apart (Figure 1A). At a high flow rate, large cells experience large inertial shear gradient lift forces, migrate across streamlines, and become stably trapped in laminar fluid microvortices that develop in the reservoirs [36]. The relatively smaller blood cells do not experience a sufficient lift force to be stably trapped and may be washed away in a solution exchange that maintains cancer cell entrapment in vortices. By lowering the flow rate, vortices dissipate to release viable cells off chip in a concentrated volume in a well plate, glass slide, or microfuge tube (∼150 μL per cycle) for downstream analysis.

Conventional PDMS fabrication processes were used to assemble devices [50]. Briefly, microfluidic channel layouts were designed using AutoCAD (Autodesk Inc.) and printed on a 20,000 dpi photomask (CAD/Art Services, Inc.). A master mold was fabricated with the mask and standard photolithographic techniques using KMPR 1050 i-line photoresist (MicroChem Corp.) on a 4-inch diameter silicon wafer (University Wafer, Inc.). PDMS was mixed in a 1:10 curing agent-to-base ratio, degassed, and cured over the mold at 65°C for 21 hours. PDMS was then cut, peeled from the wafer mold, and hole-punched (Syneo, LLC) before bonding to 3”x1” glass slides (VWR International LLC) using oxygen plasma (800 Micro RIE, Technics, Inc.) at 500 mTorr and 80 W RF power for 30 s.

### Performance testing with cell lines

MCF7 breast cancer cells (ATCC) were used to characterize device efficiency. Cells were cultured in an incubator at 37°C and 5% CO_2_ with minimum essential medium supplemented with 10% fetal bovine serum, 1% penicillin-streptomycin-glutamine, and 0.01 mg/mL human recombinant insulin (Gibco). Adherent cells were released with 0.25% (w/v) trypsin (Gibco), resuspended in media, assessed for concentration with a hemocytometer, and rocked gently on a shaker 30 min prior to experiments. Comparisons of efficiencies between devices were performed with the same batch of MCF7 cells, as cell size and deformability may fluctuate between passages and affect efficiency measurements. A target number of ∼300 cells was spiked in 4 mL of PBS or 10x diluted healthy blood and infused through the device. Flow was driven by the use of two syringe pumps (Harvard Apparatus), one for the sample solution and one for the wash solution. The device was initially primed with PBS wash solution at 8 mL/min for 30 s. Wash solution flow rate was then reduced to 1 mL/min as sample solution was infused at 7 mL/min, totaling an 8 mL/min operational flow rate for cell capture. Solution exchange was performed by returning the wash solution flow rate back to 8 mL/min while stopping the sample syringe. Cells were released from vortices by stopping the flow from the wash solution briefly to dissipate the vortices and subsequently flushing the device and tubing at a low flow rate. The enriched sample was released into a 96-well plate (Greiner CELLSTAR) for imaging and enumeration. Fluid waste was collected in a separate tube and reprocessed through the device for multiple cycles to increase capture, as specified in the results. To test viability, cells were spun down, incubated with media, and assayed with Calcein Blue AM and ethidium homodimer (Molecular Probes) every 24 hrs over the course of 4 days at which the experiment was stopped.

### Staining and enumeration

Cells collected for enumeration were fixed in 2% paraformaldehyde (Electron Microscopy Sciences) for 10 min, permeabilized in 0.4% v/v Triton X-100 (Research Products International Corp) for 7 min, blocked with 5% goat serum (Invitrogen) for 30 min, and stained with DAPI (Molecular Probes), anti CD45-phycoerythrin (CD45-PE, Clone HI30, BD Biosciences), and a fluorescein isothiocyanate (FITC)-conjugated CK cocktail against Pan-CK AE1/AE3 (eBioscience), CK3-6H5 (Miltenyi Biotec), and CK CAM5.2 (BD Biosciences) for 40 min at room temperature before imaging. The full staining protocol is included in Supplementary Material. Following CK staining, some samples were stained for granulocytes with CD66b-AlexaFluor647 (CD66b-AF647, Clone G10F5, BD Biosciences), or for EMT markers with anti EpCAM-FITC (BD Biosciences), anti vimentin-AlexaFluor647 (VIM-AF647, Abcam), and anti N-Cadherin (NCAD-AF67, Abcam). Stitched images of stained wells were acquired at 100x magnification (Zeiss Axio Observer Z1 microscope with ZEN software and Photometrics CoolSnap HQ2 CCD camera), and cells were manually enumerated by 2 different persons who were blinded to avoid bias and subsequently established consensus. For tests with cell lines, capture efficiency was calculated as the number of captured target cells over the total number of target cells spiked into the initial sample. Purity was calculated as the number of target cells collected over the total number of captured nucleated cells.

### Clinical sample processing

Blood samples were acquired in two 10 mL EDTA-coated tubes (Vacutainer, BD) from consenting stage IV lung and breast cancer patients collected from the UCLA Hematology and Oncology Santa Monica Clinic and Stanford Medical Center as well as from age-matched healthy donors following institutional review board approved protocols (UCLA IRB#11-001798 and Stanford IRB#5630). Within 4 hours of procurement, one tube of whole blood (∼8 mL per sample) was diluted 10x in PBS (∼80 mL of total volume) before processing through Vortex HT with 2 cycles, and enriched cells were collected in a 96-well plate, immunostained, imaged, and enumerated. The second tube of blood from the same patient was either processed through the previous Vortex Chip [36] or sent for analysis by the gold standard CellSearch assay for breast cancer, serviced by Quest Diagnostics. All samples were de-identified by a clinical coordinator and research staff was blinded to the sample type (between lung, breast, or healthy blood samples). CTC counts from cancer samples were compared with the maximum enumerated value from healthy samples to determine which patients have tested positive for CTCs using Vortex HT.

## SUPPLEMENTARY MATERIAL FIGURES AND TABLES


