# Supplementary material for: Classification of large circulating tumor cells isolated with ultra-high throughput microfluidic Vortex technology
Source: Oncotarget. 2016 Feb 6;7(11):12748–60. doi: 10.18632/oncotarget.7220 (PMC4914319; doi:10.18632/oncotarget.7220)
Supplement: Supplementary file 1 [file oncotarget-07-12748-s001.pdf]

Classification of large circulating tumor cells isolated with ultra-high throughput microfluidic Vortex technology

Supplementary Material

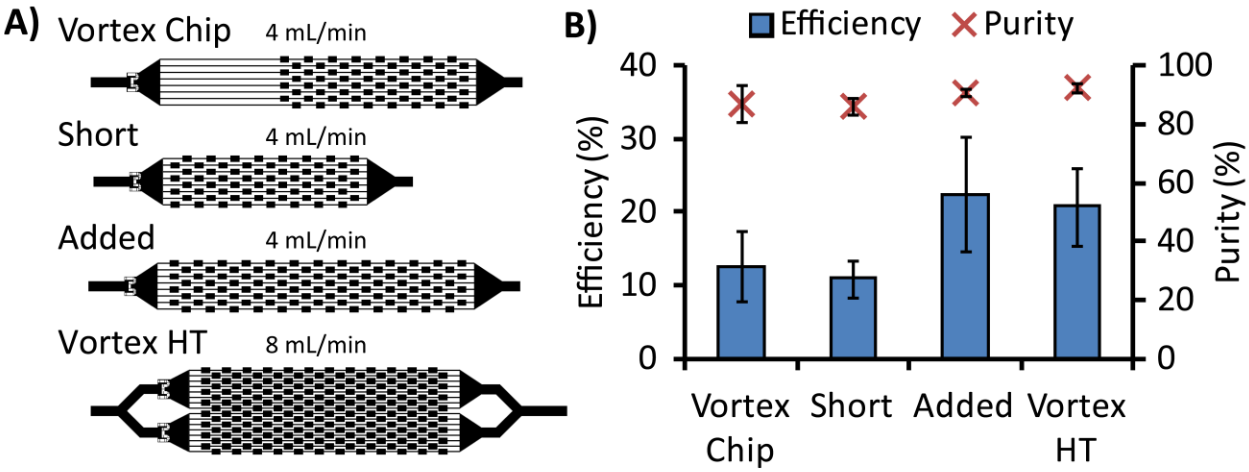

Supplementary Figure 1: *Optimization of device design*. **A)** Multiple devices were designed to test the effect of upstream channel length and the number of reservoirs, and their performance was compared to the initial Vortex device using the same protocol for efficiency testing as described in the main text. The Vortex Chip consists of long upstream channels (10 mm) followed by 8 parallel channels, each with 8 capturing reservoirs. The Short design removes focusing channels, and the Added design replaces each upstream channel with 4 more reservoirs. Vortex HT is a parallelized version of the Added design and may operate at twice the flow rate (8 mL/min). **B)** Comparison of device capture efficiencies and purities using MCF7 cells spiked in 10x diluted blood (n=3).

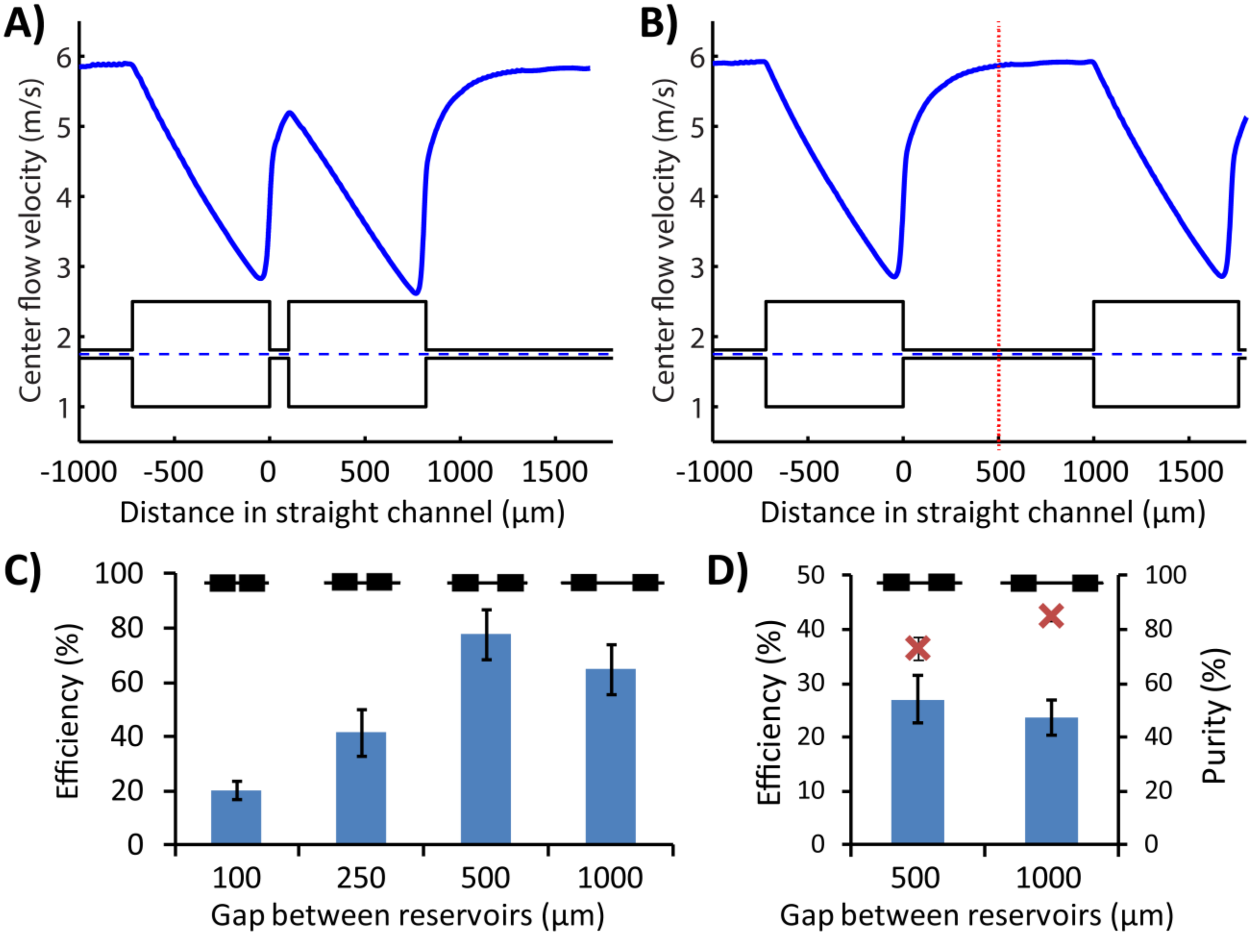

Supplementary Figure 2: *Effect of upstream channel length on particle capture.* **A)** Fluid velocity in the center of the Vortex straight channel (dashed line) does not reach a maximum over a 100 µm distance between reservoirs. **B)** However, the center velocity peaks at a minimum ~500 µm distance (dotted red line), suggesting a minimum straight distance required for flow to fully develop during sample processing. A fully developed flow profile maximizes the shear gradient lift forces required to trap particles. **C)** Tests with 20 µm diameter beads reveal a peak efficiency with a 500 µm distance upstream from each reservoir. **D)** A similar trend is shown for MCF7 cells spiked in 10X diluted blood, in which efficiency peaks at 500 µm at the cost of decreased purity. In subsequent designs, a 1000 µm distance was chosen to balance both capture efficiency and purity.

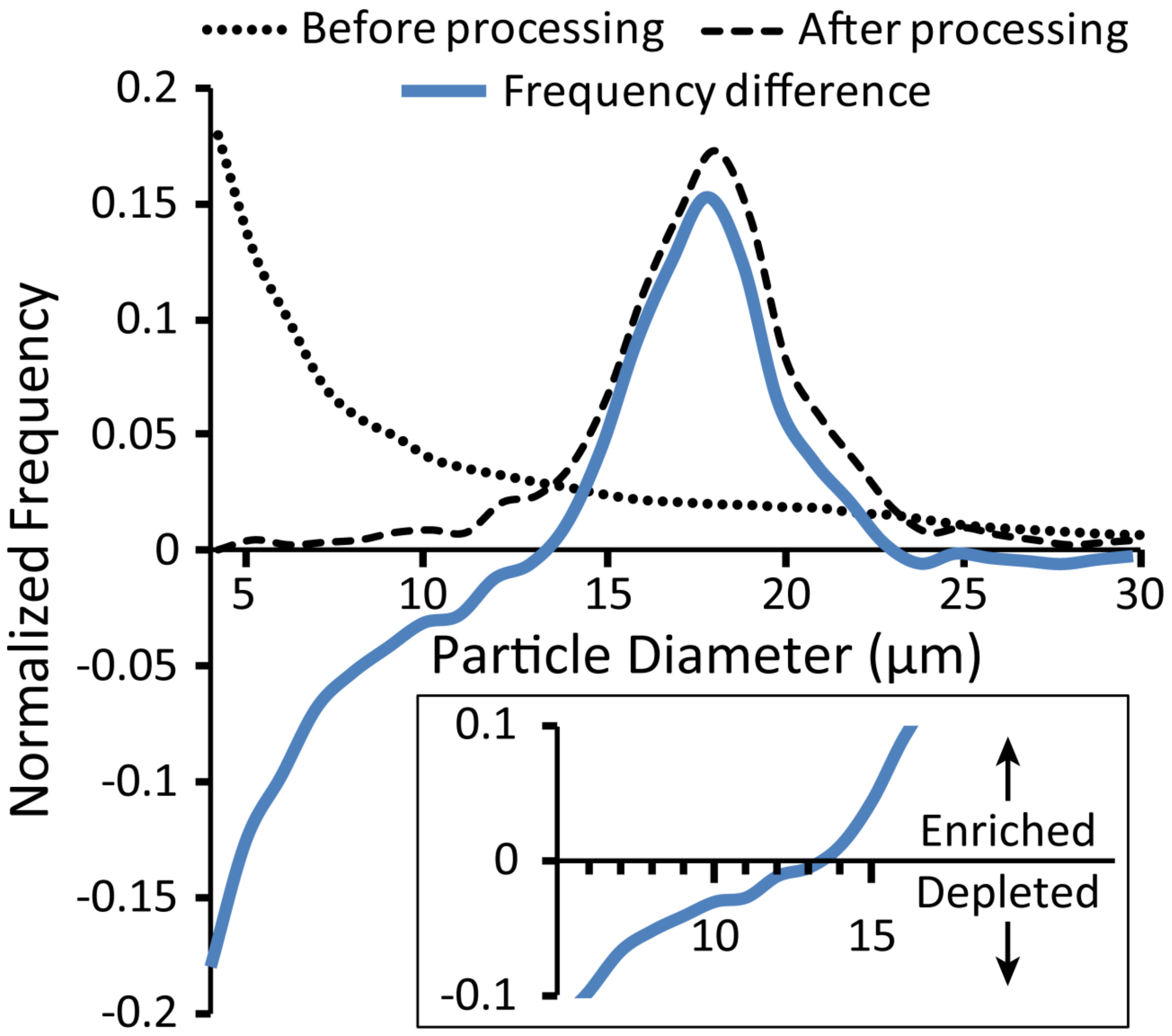

Supplementary Figure 3: *Size cutoff of Vortex HT*. Polydisperse PDMS beads (4-30 µm diameter) were processed through the Vortex HT device. The normalized frequency distribution of unprocessed beads (dotted line) was subtracted from the normalized frequency of captured beads (dashed line) to portray particle size selectivity (solid line). Bead sizes with normalized frequency differences below 0 represent size ranges which are depleted from a sample, whereas those above 0 (> ~13 µm) are favored to be captured and enriched by Vortex HT.

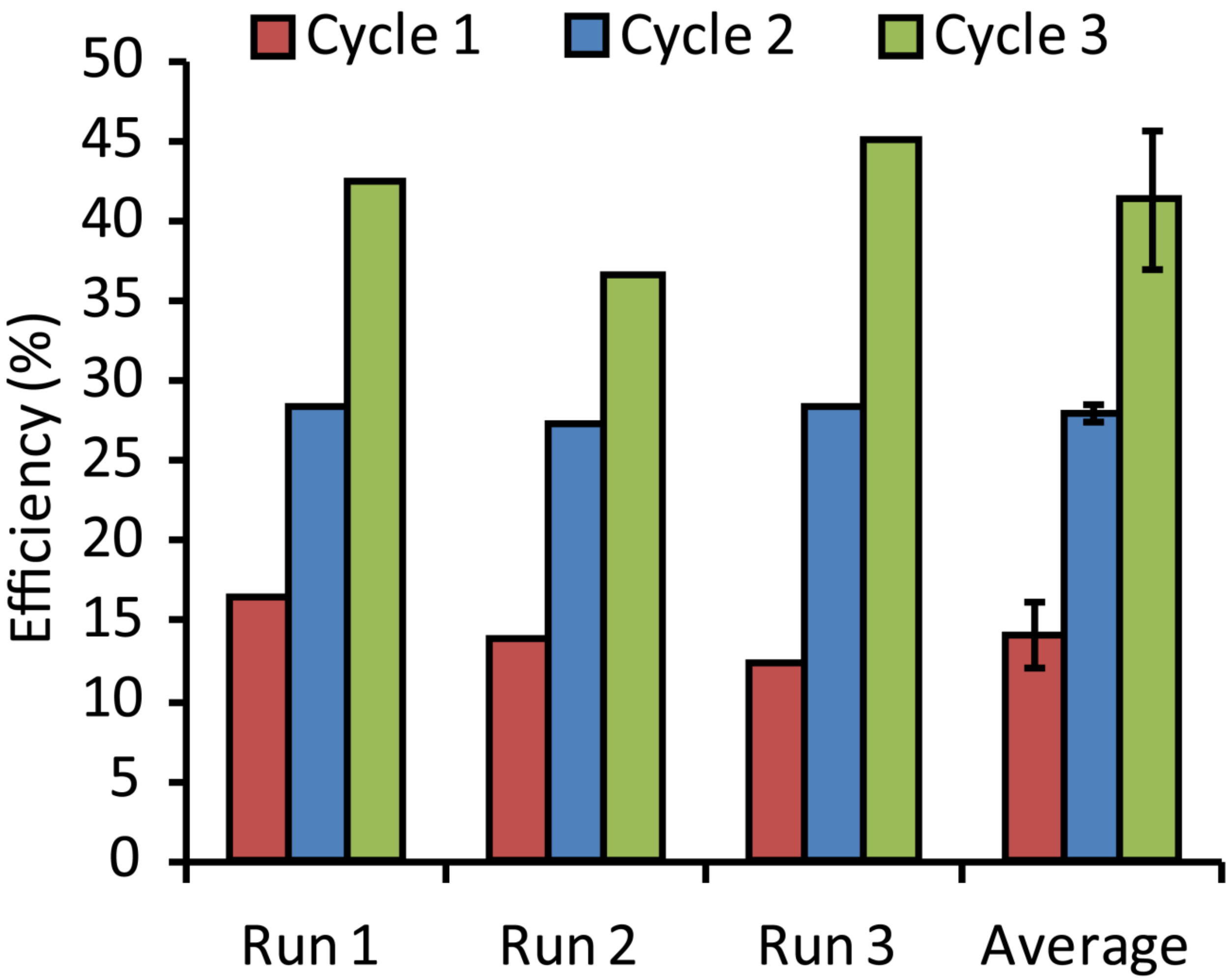

Supplementary Figure 4: *Reproducibility of sample reprocessing.* MCF7 cells spiked in blood at ~300 cells/mL were diluted 10x and processed through Vortex HT. Sample waste was collected and reprocessed for 2 more cycles. The whole procedure was repeated for 3 independent runs, each on a different PDMS device, and the cumulative capture efficiency significantly increased through all runs.

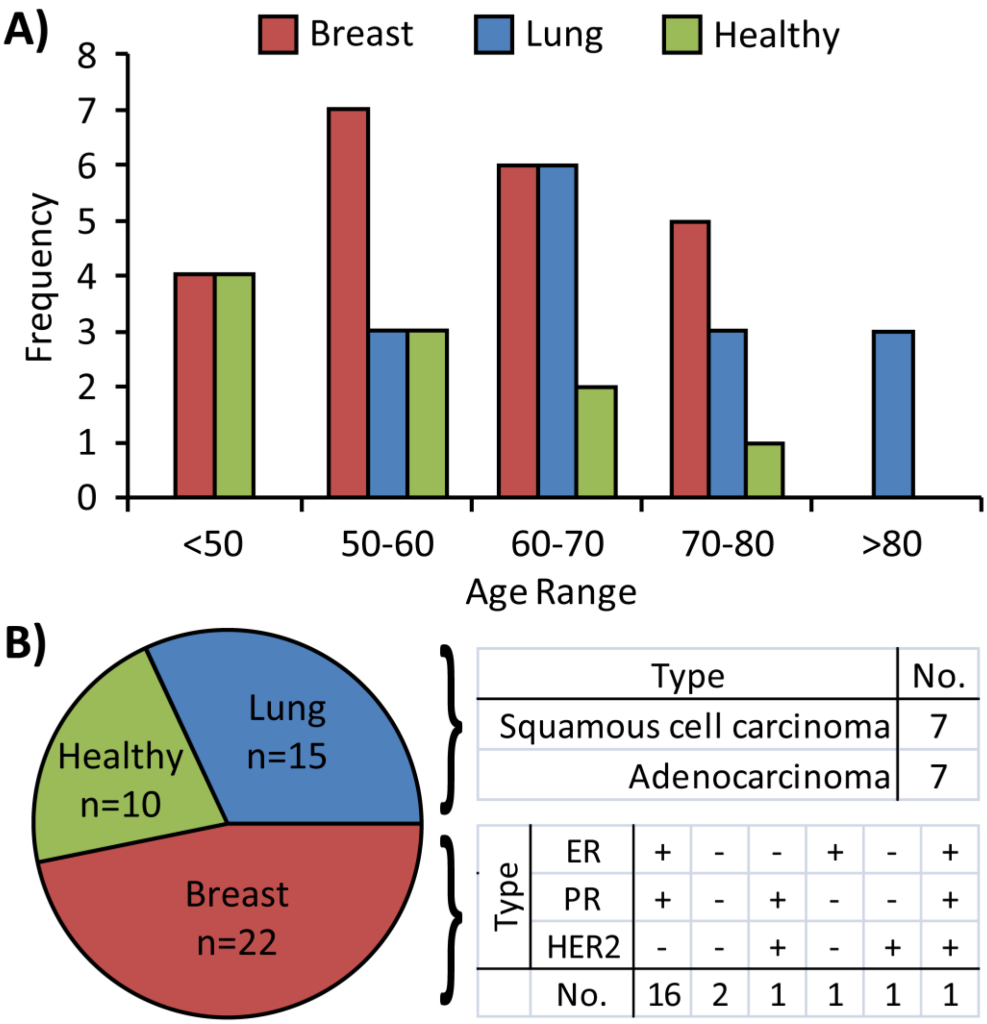

Type	No.
Squamous cell carcinoma	7
Adenocarcinoma	7

Type							
	ER	+	-	-	+	-	+
	PR	+	-	+	-	-	+
	HER2	-	-	+	-	+	+
	No.	16	2	1	1	1	1

Supplementary Figure 5: *Summary of patients enrolled in the study.* **A)** Age distribution of donors. **B)** Among the cancer samples tested, lung specimens included those categorized as squamous cell carcinoma or adenocarcinoma (information for one lung cancer patient was unknown), and breast specimens were classified as either positive or negative for estrogen receptor (ER), progesterone receptor (PR), or human epidermal growth factor receptor 2 (HER2). No strong correlations between CTC count and these parameters were found at this stage of our study (n=15 lung and 22 breast samples).

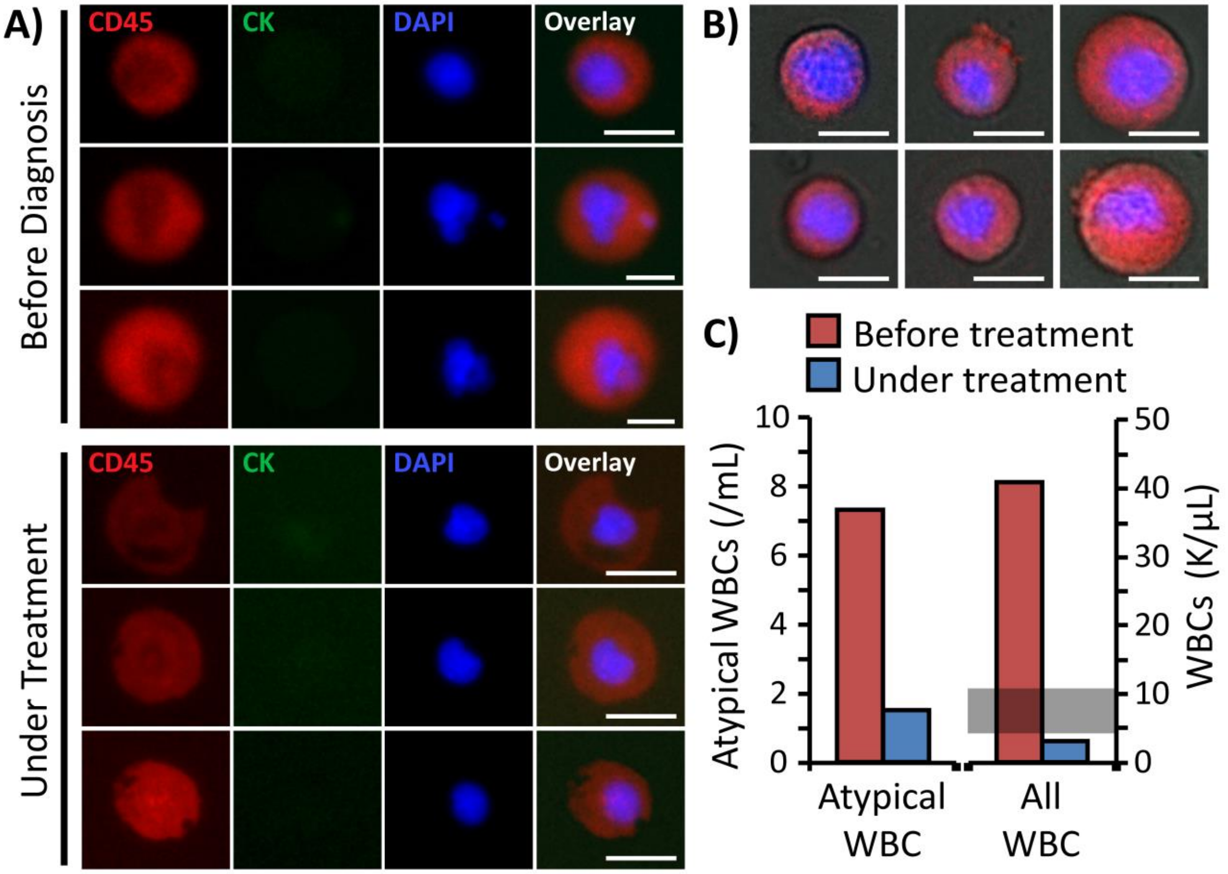

Supplementary Figure 6: *Case study of a leukemia patient*. **A)** Large atypical CD45+/DAPI+ WBCs were found in the blood from a leukemia patient. Very large atypical cells (30-40 µm) were found before the patient was diagnosed, and smaller but consistently large atypical cells (~20 µm) were found from the same patient while under treatment. **B)** All atypical WBCs exhibited large nuclei (~15 µm) but varying N:C ratios. All scale bars represent 20 µm. **C)** The number of atypical white blood cells isolated from Vortex HT decreased on the second draw after the patient started treatment. Similarly, CBC data show that the total number of WBCs decreased. The normal expected range for WBC concentration is shaded in gray.

Supplementary Table 1: Summary of all cells collected from patient blood samples.

Comparison of Vortex HT vs. Vortex Chip

No.	Type	Stage	Age & Gender	Vortex HT			Vortex Chip		
				CTC	WBC	Vol.	CTC	WBC	Vol.
1	breast	IV	75, F	26	49	6	19	18	6
2	breast	IV	69, F	23	141	6	7	66	6
3	breast	IV	48, F	7	12	6	6	7	6
4	breast	IV	77, F	73	166	6	58	151	6
5	breast	IV	71, F	29	103	6	13	41	6
6	breast	IV	75, F	45	75	6	32	35	6
7	breast	IV	51, F	19	344	6	2	22	4
8	lung	IV	66, M	21	346	6	11	411	6
9	lung	IV	67, M	39	119	6	15	34	6
10	lung	III	81, M	145	305	6	84	84	6
11	lung	IV	68, M	18	104	6	12	120	6
12	lung	IV	52, F	40	348	6	37	234	6
13	lung	IV	73, F	13	23	6	5	62	6
14	lung	IV	65, M	4	394	6	0	48	6

Comparison of Vortex HT vs. CellSearch

No.	Type	Stage	Age & Gender	Vortex HT			CellSearch	
				CTC	WBC	Vol.	CTC	Vol.
15	breast	IV	66, F	60	342	7	0	7.5
16	breast	IV	64, F	11	82	8	0	7.5
17	breast	IV	44, F	31	96	8	167	7.5
18	breast	IV	58, F	186	427	8	0	7.5
19	breast	IV	77, F	28	130	6	4	7.5
20	breast	IV	66, F	30	93	6	0	7.5
21	breast	IV	60, F	46	113	6	FAILED	7.5
22	breast	IV	63, F	9	88	8	FAILED	7.5
23	lung	IV	75, F	3	48	6	0	7.5
24	lung	IV	61, M	33	22	8	0	7.5
25	lung	IV	87, F	39	579	8	0	7.5
26	lung	IV	60, F	24	167	7	0	7.5
27	lung	IV	91, F	105	172	8	0	7.5
28	healthy	N/A	57, F	2	56	4	0	7.5
29	healthy	N/A	55, M	5	123	4	0	7.5

Processed with Vortex HT only

No.	Type	Stage	Age & Gender	Vortex HT		
				CTC	WBC	Vol.
30	breast	IV	37, F	12	119	6
31	breast	IV	59, F	20	121	6
32	breast	IV	69, F	8	307	4
33	breast	IV	46, F	3	50	4
34	breast	IV	55, F	23	740	8
35	breast	IV	58, F	67	253	6
36	breast	IV	51, F	28	106	6
37	lung	IV	52, F	6	161	8
38	lung	IV	69, M	9	11	8
39	lung	IV	76, M	28	148	6
40	healthy	N/A	20, M	0	37	2
41	healthy	N/A	23, M	2	72	6
42	healthy	N/A	35, M	5	116	6
43	healthy	N/A	69, F	6	104	6
44	healthy	N/A	77, F	2	166	6
45	healthy	N/A	41, F	4	128	6
46	healthy	N/A	69, M	1	174	6
47	healthy	N/A	60, F	3	83	6

Leukemia sample processed with Vortex HT

No.	Type	Vortex HT			
		CTC	Large WBC	WBC	Vol.
48	leukemia	8	44	460	6
49	leukemia	2	9	46	6

Immunostaining Protocol

The following procedure details the immunostaining process for a fresh suspension of CTCs in a well of a 96-well plate. The protocol requires i) fixation, ii) permeabilization, iii) blocking, and iv) staining with **Cytokeratin-FITC** (a cocktail of clones CAM5.2, CK3-6H5, and AE1/AE3), **CD45-PE** (clone HI30), and **DAPI**. Staining is also performed on wells with fixed WBCs and a CK+ cancer cell line as a control.

MATERIALS

Consumables

- Cells collected in a 96-well plate (CELLSTAR Greiner F-bottom with lid, #655-185)
- Biohazard waste container
- 20 µm filter and syringe
- Pipette tips (p10, p200, p1000)
- Tin Foil

Equipment

- Well plate centrifuge
- Pipettes (p10, p200, p1000)
- Vortexer
- Timer

Chemicals & Reagents

- Sterile PBS (without calcium and magnesium). Prepare a filtered aliquot on the day of staining
- 10% Bleach
- Triton X-100 (Sigma, #T8787-100ml)
- Deionized water
- 4% Paraformaldehyde (Electron Microscopy Sciences, 10 x 10 mL, Cat. 157-4)
- 10% Normal Goat Serum (100 mL, with 0.1% Sodium Azide, Invitrogen, # 50062Z)
- **Anti-Cytokeratin, Clone CAM5.2, FITC-conjugated, Mouse anti-human (BD # 347653)**
- **Anti-Cytokeratin, Clone CK3-6H5, FITC-conjugated, Mouse anti-human (MACS Miltenyi # 130-080-101)**
- **Anti-pan-Cytokeratin, Clone AE1/AE3, AF488-conjugated, Mouse anti-human (eBiosciences # 53-9003-82)**
- **Anti-CD45, Clone HI30, PE-conjugated, Mouse anti-human (BD Pharmingen # 555483)**
- **10 mg DAPI, dilactate, (Life Technologies # D3571)**
- Fixed white blood cells (obtained by red blood cell lysis)
- Fixed cancer cells expressing CK (HCT116 or MCF7)

PROCEDURE

Preparing Stock Reagents, BEFORE day of staining

1) 2% PFA (fixation reagent)

a) Dilute 4% paraformaldehyde with PBS 1X
b) Pass solution through a 0.2 µm filter (assume that 100 µL is lost through filter)
c) Prepare this solution under the hood. Store aliquots at 4°C

2) 0.4% Triton X-100 (permeabilization reagent)

a) Dilute 100% Triton X-100 in PBS (suggested 40 µL Triton X-100 in 10 mL PBS)
b) Pass solution through a 0.2 µm filter (assume that 100 µL is lost through filter)
c) Prepare this solution under the hood. Store aliquots at 4°C

3) DAPI at 1 mg/mL

a) Prepare the solution at 1 mg/mL in sterile water
b) Pass solution through a 0.2 µm filter (assume that 100 µL is lost through filter) to avoid the presence of any debris, which would fluoresce within the well during the imaging process
c) Divide into 1 mg/mL aliquots and store at 4°C with proper labeling

Preparing Reagents, DAY of staining

4) 5% Goat Serum (blocking reagent)

a) Dilute the 10% Goat Serum with PBS 1X
b) Pass solution through a 0.2 µm filter (assume that 100 µL is lost through filter)
c) Prepare this solution on the day of the experiment. Note that you will need 5% Goat Serum for the Blocking Step as well as for the Primary Staining Step

5) Primary Antibody Mix

a) For each well of a 96-well plate, prepare the following master mix on the day of the experiment:
   - 5 µL of CK CAM5.2-FITC (BD), i.e. ratio of 5:200
   - 5 µL of Pan-CK-FITC (MACS Miltenyi), i.e. ratio of 5:200
   - 5 µL of CK AE1-AE3-FITC (eBioscience), i.e. ratio of 5:200
   - 10 µL of CD45-PE (BD), i.e. ratio of 10:200
   - 175 µL of 5% Goat Serum
b) Multiply these volumes by the number of wells that need to be processed. Add 2 wells for WBC and CTC controls. Add an extra 10% volume to accommodate for error
c) Mix by pipetting

6) DAPI Mix

a) On the day of the experiment, for each well of a 96-well plate, prepare the following mix:
   - 1 µL of DAPI at 1 mg/mL
   - 199 µL of Sterile PBS (i.e. ratio of 1:200 for DAPI)

Immunolabeling the 96 well plate

7) Preliminary Wash

a) Each of your wells should have <300 µL worth of cell solution
b) *Centrifuge 1* (up to 2300 rpm)
c) Aspirate 200 µL from well, using sides of wall to aspirate, without touching bottom of the well, leaving a small ~50 µL liquid volume. Avoid exposing the bottom of the well to air.

8) Fixation

a) Add 2% PFA, 200 µL volume. **Incubate for 10 min at room temperature.** Stop the reaction by filling the rest of the well with PBS
b) *Centrifuge 2*. Aspirate > 200 µL (leaving ~50 µL). Add > 200 µL PBS
c) *Centrifuge 3*. Aspirate > 200 µL (leaving ~50 µL). Add > 200 µL PBS
d) *Centrifuge 4*. Aspirate > 200 µL (leaving ~50 µL)

9) Control Wells

e) Add fixed white blood cells in 1 separate well
f) Add fixed cancer cells in 1 separate well. Ideally, use a cell line expected to highly express CK, such as HCT116 or MCF7
g) *Centrifuge 5*
h) Check these control wells under the microscope to make sure you have enough cells for a relevant control before moving to the Permeabilization step

10) Permeabilization

a) Add 200 µL of 0.4% Triton X-100. **Incubate for 7 min at room temperature.** Stop the reaction by filling the rest of the well with PBS
b) *Centrifuge 6*. Aspirate > 200 µL (leaving ~50 µL). Add > 200 µL PBS
c) *Centrifuge 7*. Aspirate > 200 µL (leaving ~50 µL). Add > 200 µL PBS
d) *Centrifuge 8*. Aspirate > 200 µL (leaving ~50 µL)

11) Blocking

a) Add 200 µL of Goat Serum 5%. **Incubate for 30 min at room temperature.** Stop the reaction by filling the rest of the well with PBS
b) *Centrifuge 9*. Aspirate > 200 µL (leaving ~50 µL). Add > 200 µL PBS
c) *Centrifuge 10*. Aspirate > 200 µL (leaving ~50 µL). Add > 200 µL PBS
d) *Centrifuge 11*. Aspirate > 200 µL (leaving ~50 µL)

12) Primary Staining

a) Add 200 µL of Primary Mix. **Incubate in the dark for 40 min at 37°C.** Stop the reaction by filling the rest of the well with PBS
b) *Centrifuge 12*. Aspirate > 200 µL (leaving ~50 µL). Add > 200 µL PBS
c) *Centrifuge 13*. Aspirate > 200 µL (leaving ~50 µL). Add > 200 µL PBS
d) *Centrifuge 14*. Aspirate > 200 µL (leaving ~50 µL)

13) DAPI Staining

a) Add 200 µL of <u>DAPI Mix</u>. **Incubate in the dark for 10 min at room temperature.** Stop the reaction by filling the rest of the well with PBS
b) *Centrifuge 15*. Aspirate > 200 µL (leaving ~50 µL). Add > 200 µL PBS
c) *Centrifuge 16*. Aspirate > 200 µL (leaving ~50 µL). Add > 200 µL PBS
d) *Centrifuge 17*. Aspirate > 200 µL (leaving ~50 µL). Add > 200 µL PBS
e) *Centrifuge 18.* All cells are now at the bottom of the well for imaging

<u>Clean up</u>

- Return all equipment and supplies
- Spray & wipe down all surfaces with 70% ethanol
- Once staining and/or imaging is complete, seal the stained wells with parafilm. Wrap the well plate with tin foil to protect from light. Label the well plate with appropriate documentation and store at 4°C.
- With the collected and stained cells, proceed to imaging

IMPORTANT NOTES

1. Wear personal protective equipment. Always wear goggles, lab coat, and gloves.

2. Stain in the dark. While working with the fluorescently labeled antibodies, always make sure you are in the dark to avoid fluorophore bleaching. During incubation times, keep the well plate under tin foil.

3. Avoid cross-contamination between wells.

- Do not use the same pipette tips between the different wells, especially if the cells collected in these wells are from different patients
- Do not use the same pipette tips when removing the PBS from multiple wells during the wash steps. You may accidentally move the cells from one well to another, or contaminate cells with the wrong antibodies
- Do not collect 2 different samples in wells close to each other, as some sample may splash over the next well when there are full of liquid
- Ideally, you would collect samples in the top left, top right, bottom left, and bottom right corners of the well plate. Control cells could be located in center of the well plate
- Cover the wells with parafilm before long-term storage to avoid PBS evaporation

IMAGING

- We recommend imaging the wells using the Axio Observer Z1 microscope (Zeiss) with an automated stage. Image the entire wells of interest with a 10x objective, and perform image stitching.

Classification and Enumeration of CTCs

The following procedure is used to classify Circulating Tumor Cells (CTCs) and White Blood Cells (WBC) in immunostained well plates. It is valid for **CK-FITC**, **CD45-TRITC**, and **DAPI** stained wells. All cells must be **DAPI** positive. For each **DAPI** positive instance, move down the list in order until the conditions are met. Move to the subsequent step if unsure.

Order	Characteristic	Classification
1	Jagged shape, dark fill, or dark outline in bright-field	Debris
2	**CD45+**/**DAPI+**/CK-	WBC
3	**CK+**/**CD45-**/**DAPI+**	CTC
4	**Lobed nucleus**	WBC
5	**CK+/CD45+/DAPI+**	Favor WBC
6	**Nucleus < 9 µm**	WBC
7	**Nucleus > 9 µm, N/C < 0.80**	WBC
8	**Nucleus > 9 µm, N/C > 0.80**	CTC

EXAMPLES (All scale bars are 20 µm)

1. Jagged shape, dark fill, or dark outline in bright-field = <u>DEBRIS</u>

Debris often stains positive for a random combination of probes. Although hair-like structures are easily identified as debris, there exist pieces that may be round and similar in size as CTCs. The simplest method is to **check the morphology in bright-field.** Debris usually have i) black interiors, ii) sharp, dark outlines, or iii) very straight or sharp edges. Observe suspended cell lines to gain a sense of what cells look like.

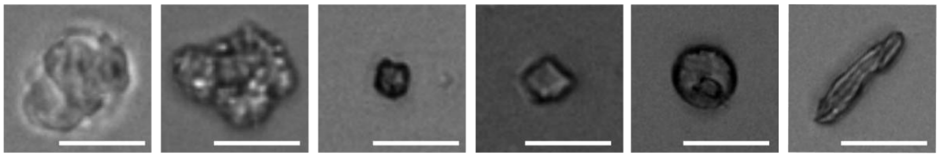

2. CD45+/DAPI+/CK- = <u>WBC</u>

This is the most straightforward and common combination of stains. For cells that are DAPI only, adjust the signal threshold in case you weak signals are missed. If it is unclear if a signal is positive or non-specific noise, compare with the noise signal from the control WBC well.

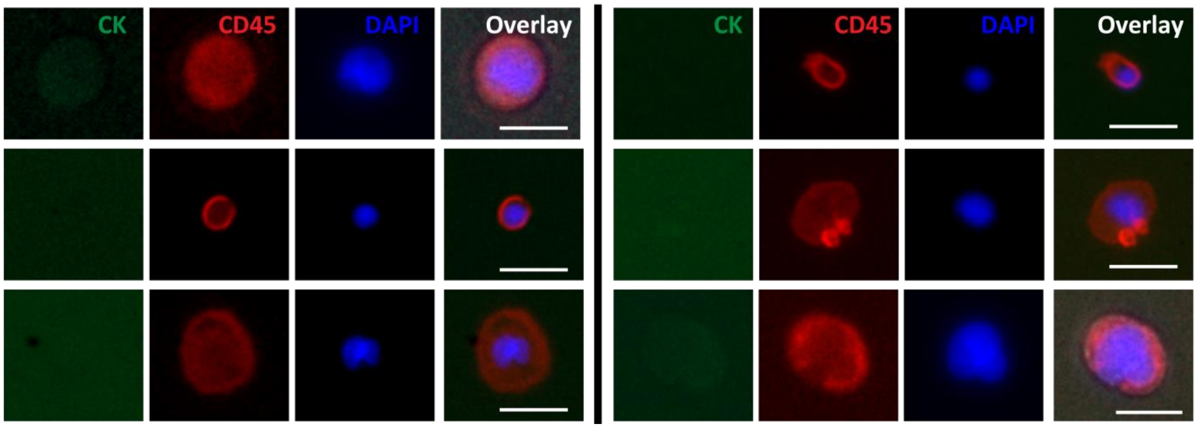

3. CK+/CD45-/DAPI+ = <u>CTC</u>

Oftentimes dust pieces will stain only CK+/DAPI+, the bright-field images much be checked for debris-like morphology. It is important to confirm the absence of a CD45 signal (due to possible double-staining) by adjusting signal thresholds.

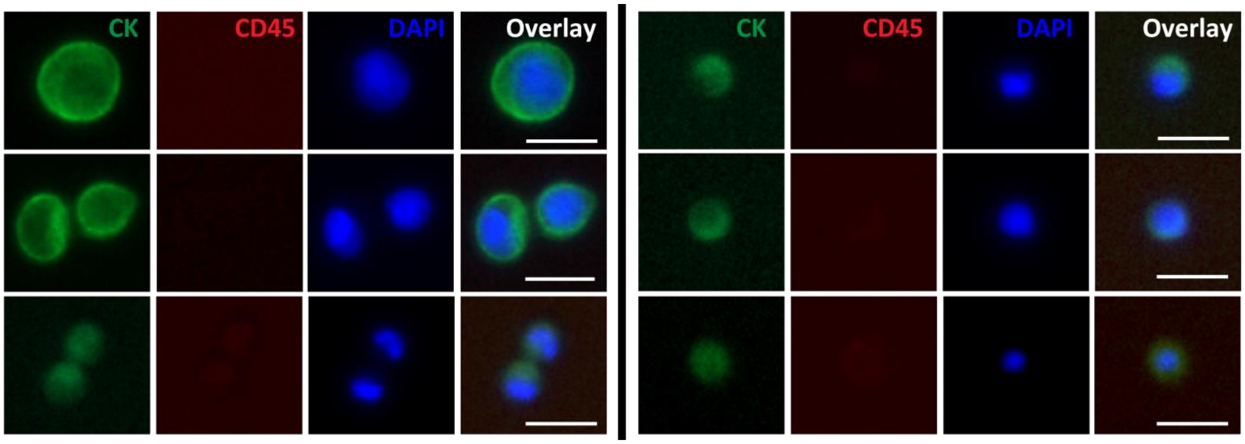

.

4. Lobed nucleus = <u>WBC</u>

For cells that are only DAPI+, the shape of the nucleus may indicate cell type. Lobed cells will usually also stain CD45+, but very weakly. Adjust the DAPI signal threshold to check if there are lobes; if the threshold is too high/saturated, it may appear spherical instead. There may exist a variety of different lobed shapes, including: "Donut", "Multinucleated", "Horseshoe", "Clover", and "Dumbell".

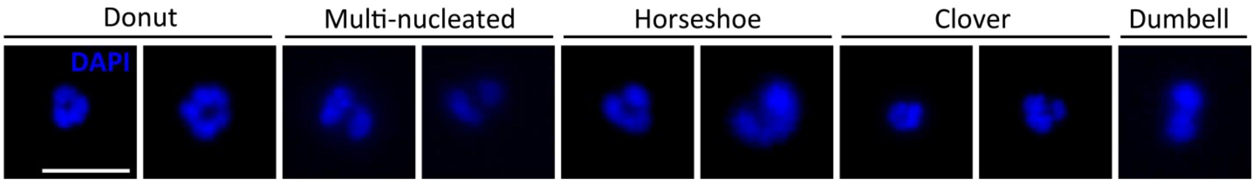

5. CK+/CD45+/DAPI+ = <u>Favor WBC</u>

Cells that are double-stained (CK+ and CD45+) are favored as WBCs. If the CD45 signal is VERY weak, the cancer cell control well should be checked -- if the residual CD45 signals are comparable, the cell is a CTC. Otherwise, the cell is a WBC, which occurs in the majority of cases. In a separate experiment, CK+/CD45+ cells were confirmed as granulocytes (which represent 35-80% of leukocytes) using a CD66b granulocyte stain.

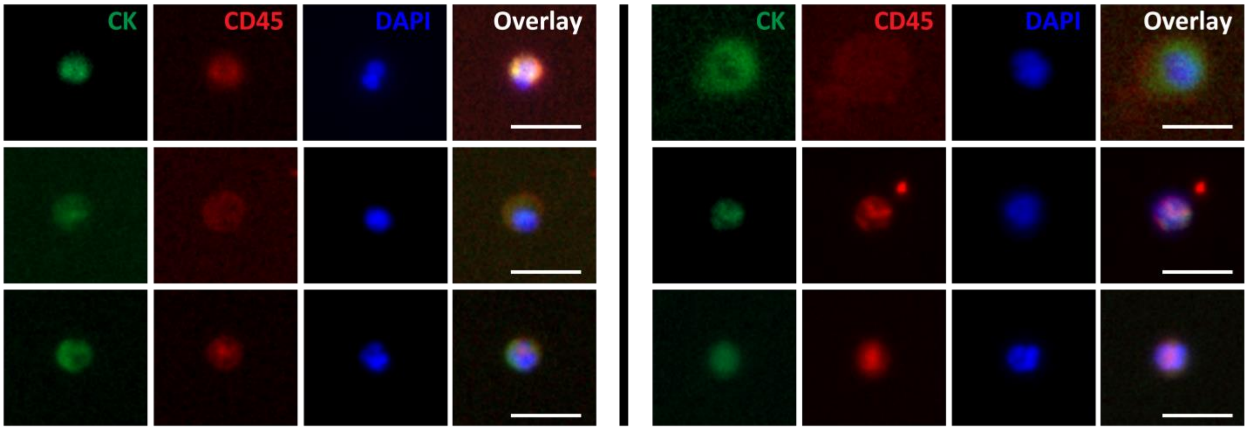

6. Nucleus < 9 μm = WBC

DAPI-only cells with small nuclei are WBCs. Cells classified in this step is rare, but this accounts for potentially lobed cells that are oriented in which the nuclei appear spherical instead of lobed, or when the focal plane is off and the nucleus is too blurred to distinguish lobes.

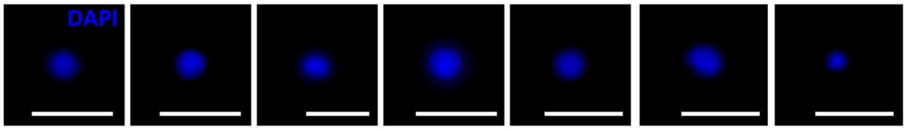

7. Nucleus > 9 μm, N/C < 0.80 = WBC

Cells with large nuclei may still be WBCs if the nuclear-to-cytoplasmic (N:C) ratio is low (<0.80). Check the N:C ratio using the DAPI and bright-field channels. These are very rare to find.

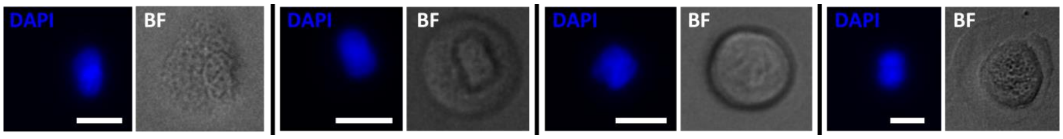

8. Nucleus > 9 μm, N/C > 0.80 = CTC

Cells with large nuclei AND high N:C ratios are CTCs. This may occur almost as frequently (~40% of cases) as finding CK+ CTCs (step 3), possibly due to low CK expression in EMT-CTCs.

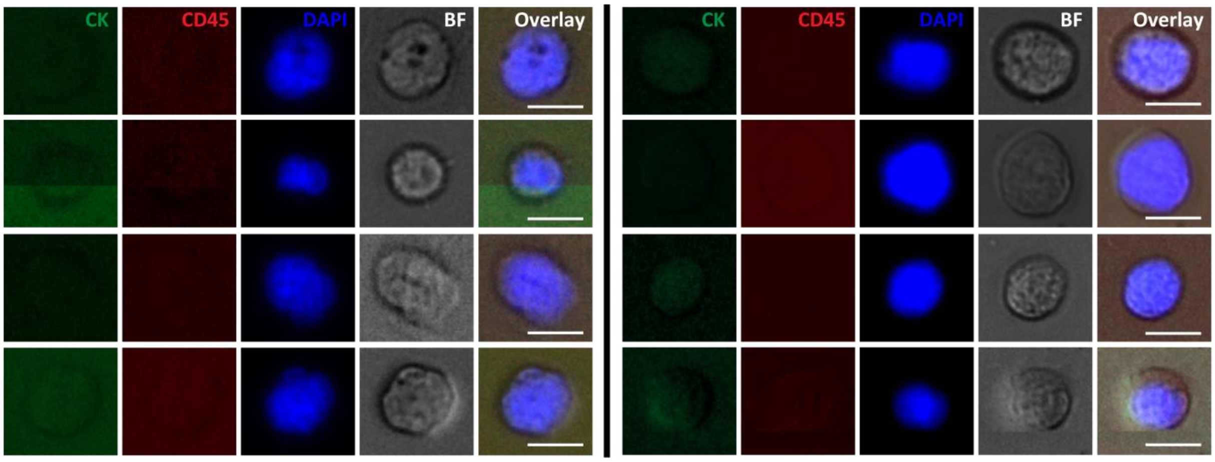

SUMMARY OF STAINING CHARACTERISTICS

<table>
<tr><th>Classification</th><th>Characteristics</th></tr>
<tr><td rowspan="2">Dust</td><td>Jagged shape</td></tr>
<tr><td>Dark under bright-field</td></tr>
<tr><td rowspan="5">White Blood Cell</td><td>DAPI+/CD45+/CK-</td></tr>
<tr><td>DAPI+/CD45+/CK+
double-stained</td></tr>
<tr><td>DAPI+/CD45-/CK-
lobed nucleus</td></tr>
<tr><td>DAPI+/CD45-/CK-
small nucleus <9µm</td></tr>
<tr><td>DAPI+/CD45-/CK-
large nucleus >9µm AND low n:c ratio <0.80</td></tr>
<tr><td rowspan="2">Circulating Tumor Cell</td><td>DAPI+/CD45-/CK+</td></tr>
<tr><td>DAPI+/CD45-/CK-
large nucleus >9µm AND high n:c ratio >0.80</td></tr>
</table>

PRACTICE EXAMPLES

Classify the following as Debris, CTC, or WBC. Scale bars represent 20 µm. Answers on next page.

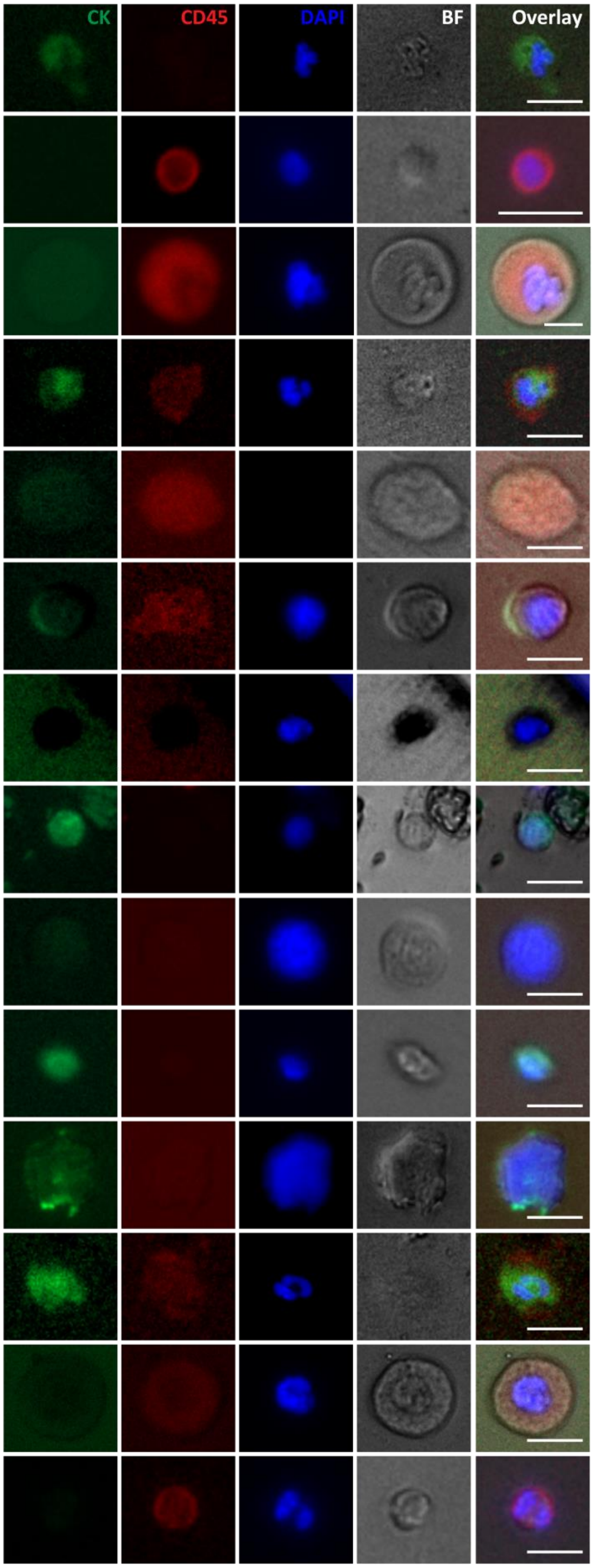

**Debris**	**CTC**	**WBC**
**Debris**	**CTC**	**WBC**
**Debris**	**CTC**	**WBC**
**Debris**	**CTC**	**WBC**
**Debris**	**CTC**	**WBC**
**Debris**	**CTC**	**WBC**
**Debris**	**CTC**	**WBC**
**Debris**	**CTC**	**WBC**
**Debris**	**CTC**	**WBC**
**Debris**	**CTC**	**WBC**
**Debris**	**CTC**	**WBC**
**Debris**	**CTC**	**WBC**
**Debris**	**CTC**	**WBC**
**Debris**	**CTC**	**WBC**

PRACTICE EXAMPLES (ANSWERS)

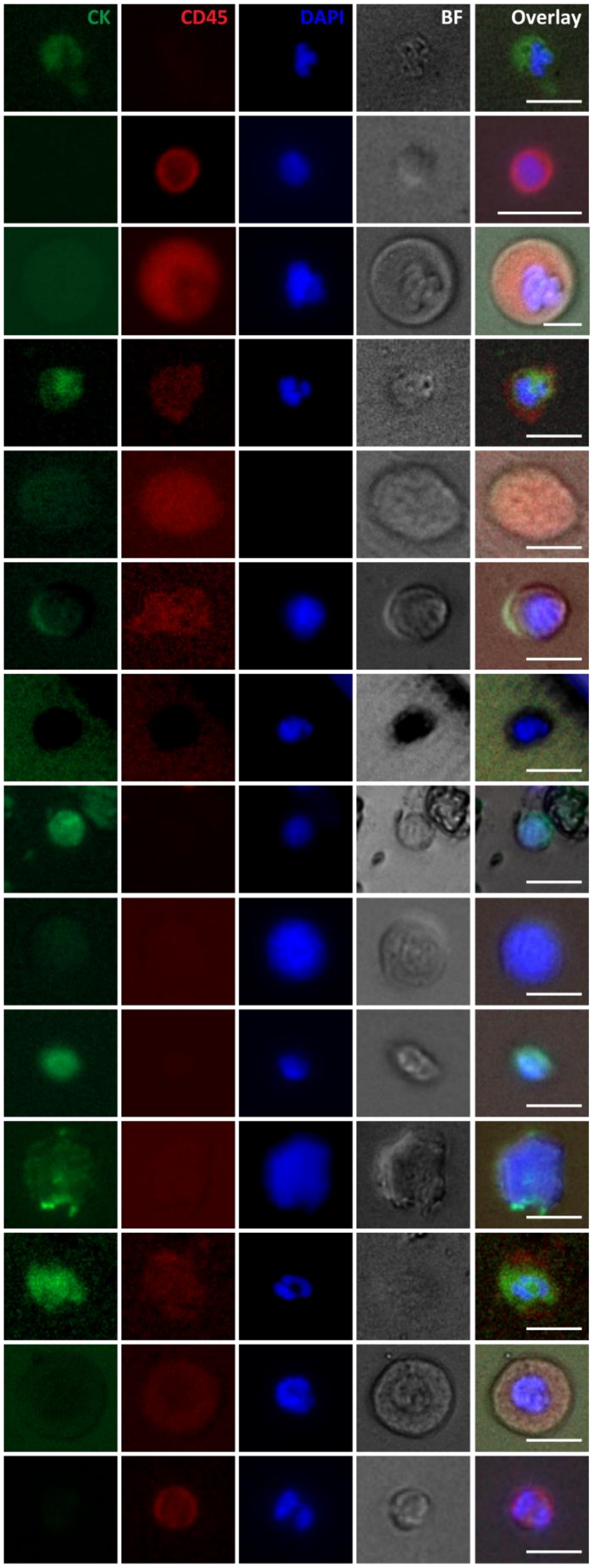

**CTC**	CK+ and DAPI+
**WBC**	CD45+ and DAPI+
**WBC**	atypical large size but CD45+ and DAPI+, and low N/C ratio
**WBC**	double-stained CK+/CD45+ and lobed DAPI nucleus
**Debris**	looks like a cell in BF but no DAPI fluorescence
**WBC**	faint CD45+ and DAPI+
**Debris**	dark-filled
**CTC**	CK+ and DAPI+, near a piece of debris
**CTC**	DAPI+ only, nucleus > 20 µm, and high N/C ratio
**CTC**	CK+ and DAPI+
**Debris**	straight & sharp edge and atypical shape/morphology
**WBC**	double-stained CK+/CD45+ and lobed DAPI nucleus
**WBC**	atypical large size but CD45+ and DAPI+, and low N/C ratio
**WBC**	CD45+ and DAPI+, and multinucleated
